# High-resolution modeling of glacier meltwater contributions to lake water level fluctuations in the Baishui River Glacier No.1 basin

**DOI:** 10.1016/j.isci.2025.113321

**Published:** 2025-08-07

**Authors:** Shoukat Ali Shah, Songtao Ai

**Affiliations:** 1 Chinese Antarctic Center of Surveying and Mapping, Wuhan University, Wuhan 430079, China; 2 State Key Laboratory of Information Engineering in Surveying, Mapping and Remote Sensing, Wuhan University, Wuhan 430079, China; 3 Key Laboratory of Polar Environment Monitoring and Public Governance, Wuhan University, Ministry of Education, Wuhan 430079, China; 4 School of Geodesy and Geomatics, Hubei Luojia Laboratory, Wuhan University, Wuhan 430079, China

**Keywords:** Earth sciences, Geology, Earth-surface processes, Glacial processes, Hydrology

## Abstract

Glacial lakes on the Tibetan Plateau are highly sensitive to cryosphere-hydrosphere interactions under climate change. This study applies a high-resolution framework to quantify sub-daily glacier meltwater contributions and hydrological impacts using Baishui River Glacier No. 1 and Blue Moon Lake Valley as a representative case. A gradient boosting machine model simulated hourly glacier melt from meteorological inputs, capturing episodic events and diurnal variability with high accuracy (RMSE = 0.0100 m/h against observed melt). Cross-correlation revealed an average 3.99-h lag between glacier melt and lake-level response, indicating delayed routing. Feature importance analysis identified air temperature and solar radiation as dominant melt drivers. Glacier meltwater contributed 87.62% of positive lake inflow, highlighting the basin’s dependence on cryospheric inputs. This integrated approach—combining high-frequency sensing, machine learning, and lag-time analysis—advances the characterization of glacier-lake dynamics and supports more accurate hydrological prediction in glacierized mountain basins.

## Highlights

• Quantifies the contribution (87.6%) of glacier melt to lake inflow during the ablation season
• GBM model accurately captures hourly glacier melt using key climate drivers
• Reveals a time-delayed meltwater transfer (3.99-h lag) from glacier to lake levels
• Framework supports sub-daily lake pulse detection and climate risk assessment

## Graphical abstract

**Figure:**
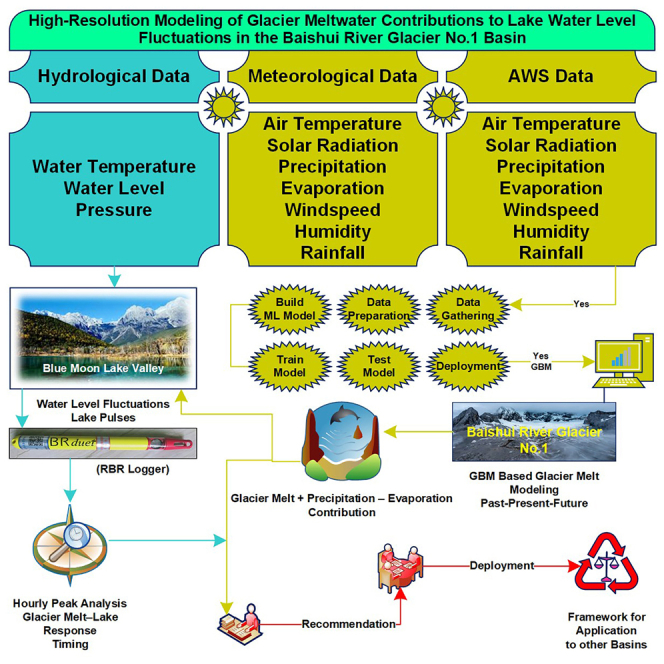

## Introduction

Understanding glacier meltwater contributions at fine temporal scales is critical for accurately predicting lake-level fluctuations in rapidly evolving high-altitude cryospheric environments. In High Mountain Asia, glacier meltwater serves as a vital hydrological buffer, particularly during dry seasons when precipitation is scarce.^1^^,^^2^ The Qinghai-Tibet Plateau, often referred to as the “Third Pole,” contains the largest ice reserves outside the polar regions and sustains major transboundary river systems such as the Yangtze, Mekong, and Brahmaputra. However, intensified atmospheric warming, altered precipitation regimes, and increased radiative forcing have led to widespread glacier mass loss across the Tibetan Plateau (TP) in recent decades.^3^ These cryospheric changes have triggered the expansion of glacial lakes, raising concerns over hydrological instability, ecosystem disruption, and the growing risk of glacial lake outburst floods.^4^^,^^5^^,^^6^

While large-scale patterns of glacier retreat and lake expansion are increasingly well documented through remote sensing and annual mass balance studies,^7^^,^^8^ a critical gap remains in understanding short-term, sub-daily glacier-lake interactions that drive rapid water level responses. Most existing research has focused on seasonal or annual timescales, with limited attention to diurnal melt cycles, hour-scale lake fluctuations, and real-time responses to transient climatic forcing. Our earlier work^5^ provided a foundational seasonal-scale assessment by quantifying glacier melt contributions over forty days using direct field observations (e.g., snow and ice depth). However, that approach lacked the temporal resolution necessary to capture intra-daily variability and lacked machine-based techniques. In contrast, the present study advances this work by introducing a high-resolution modeling framework that reconstructs past and projects future meltwater dynamics on an hourly scale using a machine learning-based gradient boosting machine (GBM) model. GBM is a powerful ensemble learning algorithm that captures complex, non-linear relationships between multiple environmental predictors and glacier melt responses. The model leverages continuous meteorological data—including air temperature, solar radiation, humidity, wind speed, precipitation, and evaporation—alongside high-frequency lake-level measurements to resolve short-term glacier–lake interactions. Unlike traditional temperature-index or degree-day models, GBM can incorporate a wide range of meteorological variables to model melt processes with greater accuracy and flexibility.^9^^,^^10^^,^^11^ The GBM model is complemented by a random forest-based attribution model that quantifies the relative contributions of glacier melt, precipitation, and evaporation to lake-level changes. Cross-correlation analyses are employed to identify time-lagged hydrological responses, revealing the temporal coupling between meltwater generation and lake-level fluctuations. These water level fluctuations—we proposed the name lake pulses—represent subtle, high-frequency variations in lake levels that are often missed in conventional monitoring but are critical for understanding glacier-lake feedbacks. Detection of these pulses is made possible through high-precision RBR Duet loggers, which provide continuous, sub-hourly lake-level data.^5^

A key innovation of this study lies in the transferability of the proposed framework. Recognizing that similar glacier-lake dynamics occur across other high-altitude basins; the framework is designed to be scalable and adaptable. By integrating machine learning, *in-situ* monitoring, and climatic attribution, it enables application in data-rich and data-sparse regions alike. This generalizability is particularly valuable for regions where glacial lakes are expanding but observational infrastructure is limited. The Yulong Snow Mountain (YSM) region, located on the southeastern margin of the TP, serves as an ideal testbed for this approach. Home to the southernmost temperate glaciers in mainland Eurasia, YSM is influenced by both Southeast Asian monsoon systems and high-altitude climatic regimes.^12^^,^^13^ The Baishui River Glacier No. 1—a rapidly retreating maritime glacier—feeds directly into Blue Moon Lake Valley, a small, sensitive proglacial lake system. This study shows how glacial lakes respond to climate shifts by tracking meltwater contributions throughout the day and pinpointing delays caused by runoff, subsurface flow, and short-term storage near the glacier, presenting practical insight for early warnings, flood planning, and managing water resources. The specific objectives of this study are to: (1) quantify and predict direct glacier melt contributions to lake-level variability at hourly scales; (2) determine the time-lagged hydrological responses of the lake to glacier melt, precipitation, and evaporation; and (3) develop a transferable, high-resolution glacier-lake-climate interaction framework applicable to other catchments across the TP. By linking fine-scale glacial melt events to immediate hydrological responses, this study refines existing modeling approaches and introduces a high-resolution, integrated framework that supports climate sensitivity analysis, forecasting, and water resource planning in glacierized mountain regions.

## Results

### Variability and influence of meteorological inputs on glacier melt modeling

The meteorological inputs incorporated into the GBM model exhibited substantial variability, reflecting the dynamic atmospheric conditions influencing glacier melt at Baishui River Glacier No.1. Air temperature, a primary driver of surface energy exchange, averaged 5.69°C, with extremes ranging from −13.01°C to 20.13°C, indicating frequent transitions between freezing and melt-favorable conditions. Incoming solar radiation, another critical energy source, varied widely from −7.21 to 1082 W/m^2^, with a mean of 164.03 W/m^2^, pointing out the importance of radiative forcing in modulating melt intensity (Table 1). Precipitation was generally sparse, averaging 0.12 mm/h, though episodic rainfall events reached up to 20 mm/h, potentially contributing to melting through latent heat exchange during rain-on-snow occurrences. Additional atmospheric parameters included relative humidity (mean: 74.36%), wind speed (mean: 0.95 m/s), and atmospheric pressure (mean: 674.18 hPa), each influencing surface energy fluxes and boundary layer dynamics to varying degrees. Crucially, the inclusion of interaction terms significantly enhanced the model’s ability to capture nonlinear melt responses. The temperature-solar radiation interaction exhibited a mean value of 1646.31, with a broad range from −1807.63 to 19526.40 (Table 1), highlighting the amplified melt potential under simultaneous high-temperature and high-radiation conditions. In contrast, the temperature-rainfall interaction, though less prominent (mean: 1.34; max: 286.80), suggests that under specific thermal and hydrological scenarios, rainfall can act as a secondary but episodically important melt accelerator.

**Table 1:** Statistics summary of glacier melting, and climate parameters

Parameters	Mean	Min	25%	50%	75%	Max	Std	Median
Observed glacier (m/h)	0.000	−0.032	−0.001	0.000	0.001	0.034	0.007	0.000
Temperature (°C)	5.690	−13.010	0.282	6.804	11.613	20.130	7.183	6.804
Solar radiation (W/m^2^)	164.028	−7.209	−1.882	0.316	257.475	1082.000	258.689	0.316
Rainfall (mm)	0.124	0.000	0.000	0.000	0.000	20.000	0.689	0.000
Humidity (%)	74.357	2.257	56.290	82.000	97.300	100.000	25.086	82.000
Windspeed (m/s)	0.954	0.000	0.000	0.322	1.624	6.193	1.239	0.322
Pressure (hPa)	674.184	668.024	672.839	674.407	675.567	680.530	1.982	674.407
Temperature -solar radiation	1646.310	−1807.628	−2.703	12.147	2037.366	19526.400	3165.479	12.147
Temperature -rainfall	1.337	0.000	0.000	0.000	0.000	286.800	8.444	0.000
GBM-modeled glacier melt (m/h)	−0.001	−0.032	−0.004	0.000	0.001	0.034	0.007	0.000

### GBM-based modeling of glacier melt: Calibration, validation, and prediction

**Figure 1:**
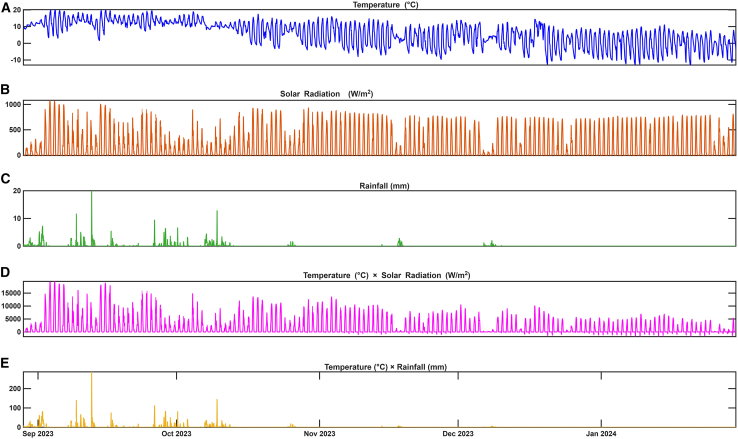
Temporal variability of meteorological drivers and their interactions influencing glacier melt at Baishui River Glacier No. 1 (A) hourly air temperature, (B) incoming solar radiation, (C) rainfall, (D) temperature-solar radiation interaction term, and (E) temperature-rainfall interaction term.

**Figure 2:**
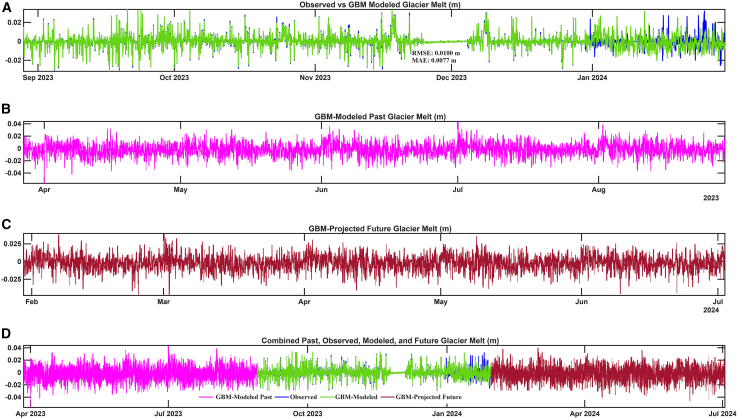
Comparison of observed, GBM-modeled, and GBM-projected glacier melt dynamics at Baishui River Glacier No. 1 (A) Observed vs. GBM-modeled hourly melt rates (August 2023–February 2024), (B) GBM-modeled Past melt rates (March–September 2023), (C) GBM-projected Future melt rates (February–July 2024), and (D) Integrated timeline combining observed, modeled, and future predicted melt trends (March 2023–July 2024).

A GBM model was applied to simulate hourly glacier melt rates over Baishui River Glacier No.1 during the observational period (August 2023 to February 2024). Key meteorological variables—near-surface air temperature, incoming solar radiation, precipitation, relative humidity, wind speed, and atmospheric pressure—served as predictors, along with interaction terms such as temperature-solar radiation and temperature-rainfall (Figures 1A–1E). The model was calibrated using observed hourly meltwater measurements and validated by comparing modeled melt rates against independent observed data. Validation statistics indicated a strong agreement between observed and GBM-predicted melt rates, supporting the model’s ability to capture the temporal variability of glacier melt. Subsequent analysis demonstrated the model’s capacity to reconstruct current and past melt events and project future melt trends (Figures 2A–2D; Table 1). The obtained RMSE (0.0100 m/h) and mean absolute error (MAE) (0.0077 m/h) values indicate a high level of predictive accuracy, effectively replicating observed melt dynamics, including transient refreezing phases and abrupt melt intensifications. The GBM model effectively captured and predicted melt rates had a mean of −0.001 m/h, a median of 0 m/h, and ranged from a minimum of −0.032 m/h (indicating brief refreezing events) to a maximum of 0.034 m/h, with a standard deviation of 0.007 m/h (Table 1). These statistics suggest that while average melt rates were low, episodic high melt events occurred. Observed melt values showed a similarly narrow distribution, with a mean of 0 m/h and a maximum of 0.034 m/h, supporting the presence of limited but frequent melt episodes. The close agreement between observed and GBM-modeled values, particularly in capturing the magnitude and timing of melt events (Figures 2A and 2D), points out the robustness of the GBM approach. Time series analysis (Figures 1A and 1B) revealed that peak melt events coincided with periods of high solar radiation and positive air temperatures. This pattern highlights the dominant role of short-term meteorological drivers—such as diurnal temperature fluctuations and episodic solar radiation peaks—in controlling meltwater generation. These findings are consistent with previous observations over maritime glaciers in the southeastern TP,^8^^,^^14^ where frequent and rapid changes in weather conditions have been shown to exert strong control over surface melt dynamics. Furthermore, the GBM model demonstrated strong predictive capability for both retrospective and prospective glacier melt estimation. As illustrated in Figure 2, panel (B) presents reconstructed melt rates for the past ablation period (March–September 2023), while panel (C) shows projected melt rates ahead (February–July 2024). Panel (D) integrates observed, GBM-modeled, and GBM-projected melt trends across a continuous timeline (January 2023–July 2024), highlighting the model’s utility in capturing seasonal transitions, episodic melt events, and potential future anomalies under evolving climatic conditions.

### Feature importance analysis of glacier melt rate predictors

**Figure 3:**
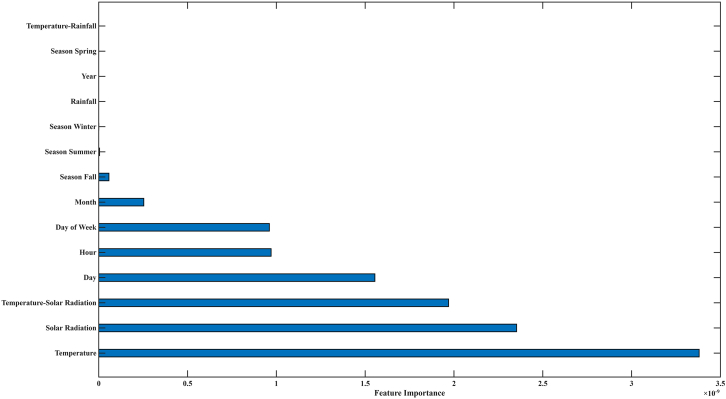
Feature importance plot highlighting key climatic influences on Baishui River Glacier No.1 during the observation period

The feature importance analysis derived from the GBM model presents quantitative insight into the relative contribution of each predictor to glacier melt rate estimation. Among all variables (Figure 3), near-surface air temperature exhibited the highest importance score (3.38 × 10^−9^), affirming its dominant role in modulating surface energy balance and melt processes. This result is consistent with established glaciological theory, where temperature is a primary driver of ablation through its direct influence on sensible heat flux and phase change dynamics.^15^ Solar radiation (2.35 × 10^−9^) and the temperature-solar radiation interaction term (1.97 × 10^−9^) were also among the top contributors, highlighting the synergistic effect of radiative and thermal energy in enhancing melt. Therefore, these results depict the importance of shortwave energy input, particularly under warm atmospheric conditions, in accelerating ice loss. Temporal variables such as day (1.55 × 10^−9^), hour (9.70 × 10^−10^), and day of the week (9.61 × 10^−10^) demonstrated moderate importance, suggesting that diurnal and weekly cycles introduce measurable variability in melt behavior, likely due to fluctuations in solar angle and atmospheric stability. These results highlight the critical role of shortwave radiation—especially under warm atmospheric conditions—in accelerating ice melt. Temporal variables such as day (1.55 × 10^−9^), hour (9.70 × 10^−10^), and day of the week (9.61 × 10^−10^) showed moderate importance, indicating that diurnal and weekly cycles contribute to melt variability, likely due to changes in solar angle and atmospheric stability. Monthly and seasonal indicators (month: 2.53 × 10^−10^; season-fall: 5.72 × 10^−11^) contributed marginally, reflecting broader climatological trends but with less predictive power at the hourly scale. In contrast, rainfall, year, season-spring, and the temperature-rainfall interaction term all registered zero importance, indicating negligible influence on melt prediction within the model framework. This suggests that rain-on-snow events and interannual variability were either infrequent or had limited impact during the observation period. The analysis confirms that glacier melt is predominantly governed by short-term meteorological conditions—particularly temperature and solar radiation—while seasonal and hydrological variables play a secondary role. These results align with prior studies on maritime and monsoonal glaciers in the southeastern TP,^8^^,^^16^ reinforcing the sensitivity of glacier melt to immediate atmospheric forcing. The limited importance of seasonal variables such as month and season-fall reflects the dominance of immediate meteorological conditions over broader climatological cycles during the late ablation season.

### Temporal dynamics between glacier melting and lake water level fluctuations

**Figure 4:**
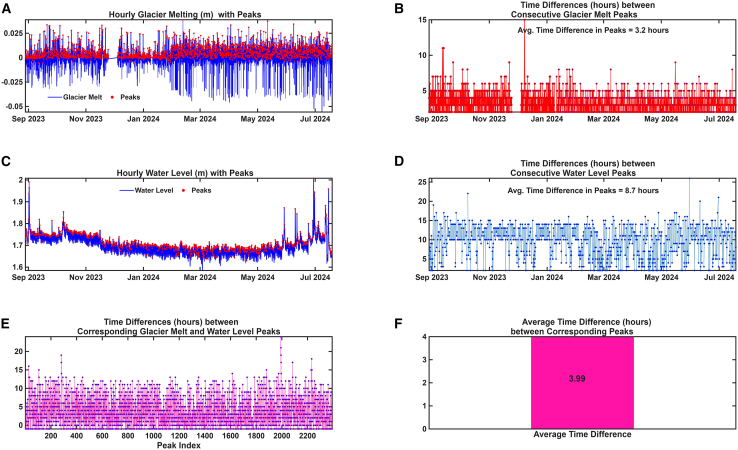
Time-series analysis of glacier melt and lake water level fluctuations (A and B) Hourly glacier melt rates show the timing and frequency of peak melt events. (C and D) Observed lake water level variations highlight the delayed response relative to glacier melt. (E and F) Estimated travel time of meltwater from Baishui River Glacier No. 1 to Blue Moon Lake Valley, based on peak-to-peak time lag analysis.

The analysis of peak events revealed distinct temporal patterns between glacier meltwater generation and downstream lake water level responses. The average time difference between consecutive glacier melt peaks was approximately 3.2 h (Figures 4A and 4B), indicating a relatively rapid recurrence of meltwater production events driven by short-term atmospheric forcing, such as diurnal solar radiation cycles. In contrast, the average time difference between consecutive water level peaks in the lake was approximately 8.7 h (Figures 4C and 4D), reflecting a more damped and delayed hydrological response compared to the glacier’s immediate surface melting behavior. This temporal offset suggests that although melting is sensitive to high-frequency climatic variations, the downstream lake system integrates and buffers meltwater inflows over longer timescales. The observed average time lag of ∼3.99 h (Figures 4E and 4F) between glacier melt peaks and lake water level responses corresponds to an average meltwater travel velocity of ∼0.7 m/s over the ∼10 km distance from the glacier terminus to the lake. This is consistent with typical flow velocities in steep, glacier-fed alpine catchments, where routing involves a combination of surface and subsurface pathways, temporary storage, and channelized flow.^17^

### Statistical influence of glacier melts and atmospheric variables on lake dynamics

**Figure 5:**
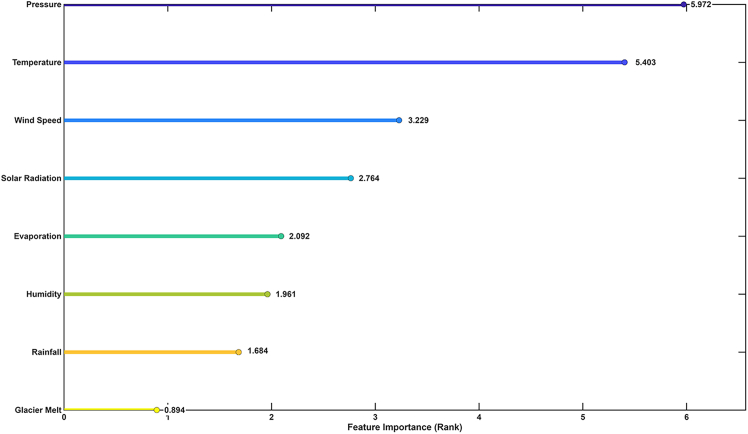
The relative feature importance rank of hydrological drivers for water level fluctuations in the Blue Moon Lake Valley

The analysis of hydrological drivers influencing Blue Moon Lake’s water level dynamics revealed distinct patterns in predictive importance over the observation period (Figure 5). Atmospheric pressure (5.973) and air temperature (5.403) emerged as the most influential predictors, likely due to their combined effects on evaporation and glacier melt processes. Wind speed (3.229) and solar radiation (2.764) also played significant roles by enhancing surface energy exchange. Evaporation (2.092) and humidity (1.961) contributed moderately, reflecting their roles in longer-term water balance regulation. Rainfall (1.684), though limited in volume, showed high predictive importance, indicating its role in triggering short-term lake level spikes. Glacier melt, with a lower importance score (0.894), had a more modest statistical influence, though it remains volumetrically significant in alpine catchments where seasonal melt pulses are common.

### Volumetric attribution of positive water inputs and lake response

**Figure 6:**
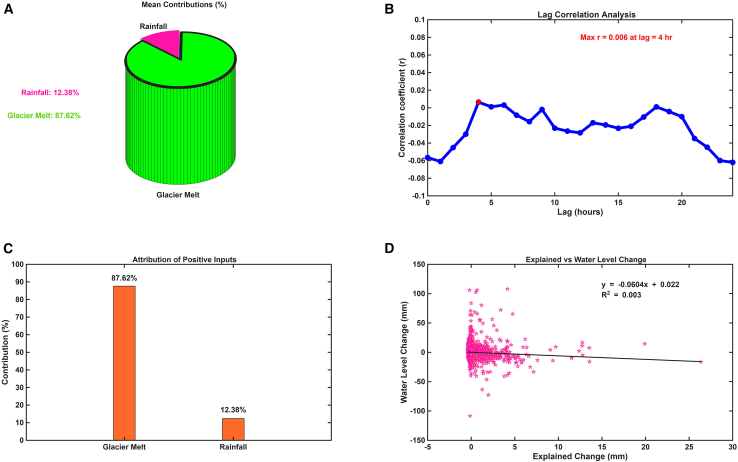
Positive water input attribution in the Blue Moon Lake Valley (A) mean contribution showing percentage (B) lag correlation (C) attribution of positive input, and (D) explained vs. water level change.

The hydrological source attribution analysis for the lake basin indicated that glacier meltwater was the dominant source of positive water inputs during the observation period. Quantitatively, glacier melt accounted for approximately 87.62% of the total inflow, whereas direct rainfall contributed only 12.38% (Figures 6A and 6C). These proportions were calculated by excluding evaporation, which was treated as a loss process and therefore omitted from the input-only framework. This stark imbalance highlights the basin’s strong reliance on cryospheric processes as the principal driver of hydrological gains. To examine how these inputs influenced lake-level dynamics, a correlation analysis was performed between combined water inputs (glacier melt plus rainfall minus evaporation) and the observed hourly lake-level changes. A lagged correlation analysis, previously described in the temporal dynamics section, was employed to assess whether a delayed lake response could account for the weak correlation. The results showed only a marginal improvement at a 4-h lag (r = 0.006), as shown in Figure 6B. This lag is consistent with the physical distance (∼10 km) between the glacier terminus and the lake. Earlier findings indicated that glacier melt peaks approximately 2.77 h after peak solar radiation, whereas the lake level peaks about 7.8 h later, yielding an average delay of 3.99 h. This supports the notion that the lake’s response is delayed due to the travel time of meltwater through the valley system. However, even when accounting for this lag, the correlation remained negligible, indicating that hourly-scale lake-level variations are not strongly governed by immediate hydrological inputs. Further assessment using linear regression between modeled net input flux and observed lake-level change (Figure 6D) produced a regression line of y = −0.0604x + 0.022 and a very low coefficient of determination (R^2^ = 0.003). This suggests that the combined hydrological inputs explained only a minimal fraction of the observed variability in lake level. These findings imply that other unmeasured or temporally delayed processes, such as subsurface flow, internal basin storage, or groundwater exchange, may significantly influence the lake’s short-term response to meltwater and rainfall inputs. The results highlight the complex hydrological behavior of the glacier-lake system, where dominant water sources do not necessarily translate into immediate or observable lake-level changes at fine temporal resolutions.

## Discussion

This study presents a high-resolution, machine learning-driven framework for quantifying sub-daily glacier meltwater contributions and their hydrological impacts on proglacial lakes, using Baishui River Glacier No. 1 and its downstream Blue Moon Lake Valley as a representative case. The application of the GBM model proved highly effective in capturing the temporal variability of glacier melt at hourly resolution. Performance metrics (RMSE = 0.0100 m/h; MAE = 0.0077 m/h) indicate strong agreement with observed melt rates, including the detection of brief refreezing events and episodic high-melt episodes. These results align with recent studies demonstrating the utility of ensemble learning models in simulating complex hydrological processes in mountainous terrain.^18^

A key contribution of this study is the identification and quantification of lagged lake-level responses to glacier meltwater pulses. The average lag time of 3.99 h between melt peaks and lake-level responses corresponds well with the ∼10 km distance between the glacier terminus and the lake. This delay reflects the time required for meltwater to travel through the valley system and is consistent with previous findings in glacierized basins, where runoff is often routed through subsurface or delayed surface pathways.^19^^,^^20^ Our earlier work^5^ using forty days of high-frequency data also supports this lag estimate.

Despite glacier melt accounting for nearly 87.62% of the total positive inflow, its predictive importance in explaining short-term lake-level fluctuations was notably low. This distinction between volumetric contribution and predictive influence is critical. While glacier melt overwhelmingly dominates the water budget, it does not translate directly into immediate or proportionate changes in lake level. The weak correlation between combined hydrological inputs (glacier melt, rainfall, and evaporation) and observed lake-level changes (R^2^ = 0.003), even after applying a 4-h lag, indicates a decoupling between input fluxes and surface water response. This low correlation suggests that the lake does not respond instantaneously or linearly to external inputs, likely due to internal basin processes such as subsurface infiltration, temporary storage in soil or snowpack, delayed routing through channel networks, and groundwater exchange. Such findings align with previous studies that have emphasized the moderating role of basin morphology, sediment permeability, and subsurface hydrology in shaping lake-level dynamics in alpine catchments.^21^

The feature importance analysis further reinforces the physical realism of the GBM model. Near-surface air temperature and solar radiation emerged as the most influential predictors of melt, consistent with established glaciological theory.^15^ The negligible influence of rainfall and seasonal indicators suggests that melt processes during the study period were primarily thermally driven, with limited contribution from rain-on-snow events or interannual variability. This is particularly relevant for temperate monsoon-influenced glaciers like Baishui River Glacier No. 1, where shortwave radiation and warm air advection are dominant melt drivers. Although seasonal variables representing winter and summer periods were included in the model, their feature importance scores were low. This does not imply that seasonal cycles are irrelevant to melt processes, but rather reflects the fine temporal resolution and the relatively narrow time window of the study, which spanned primarily the late ablation season. During this period, short-term variability in temperature and solar radiation dominated melt behavior, reducing the explanatory power of broader seasonal categories. Temperate monsoon-influenced glaciers such as Baishui River Glacier No. 1 often experience melt events outside conventional summer months due to their lower elevation and exposure to humid, warm air masses. Thus, for high-frequency melt prediction, immediate meteorological forcing outweighs long-term climatological labels such as summer or winter. However, for longer-term or interannual modeling, these seasonal indicators might gain more significance.

Compared to traditional temperature-index or degree-day models,^22^^,^^23^^,^^24^ the GBM approach presents several advantages. It accommodates multivariate inputs and their interactions, captures non-linear dependencies, and remains robust under varying data availability conditions. This flexibility is fundamental for application in data-sparse regions of the TP, where ground-based observations are limited. Moreover, the integration of high-frequency lake-level monitoring using RBR Duet loggers enables real-time validation and calibration of model outputs, bridging the gap between process-based modeling and empirical observation. Overall, this study demonstrates the value of combining machine learning, high-frequency monitoring, and physical hydrology to better understand glacial lake systems. The dual perspective—linking physical water fluxes with statistical explanatory strength—is important for anticipating future changes in alpine hydrology under evolving climatic conditions.

**Figure 7:**
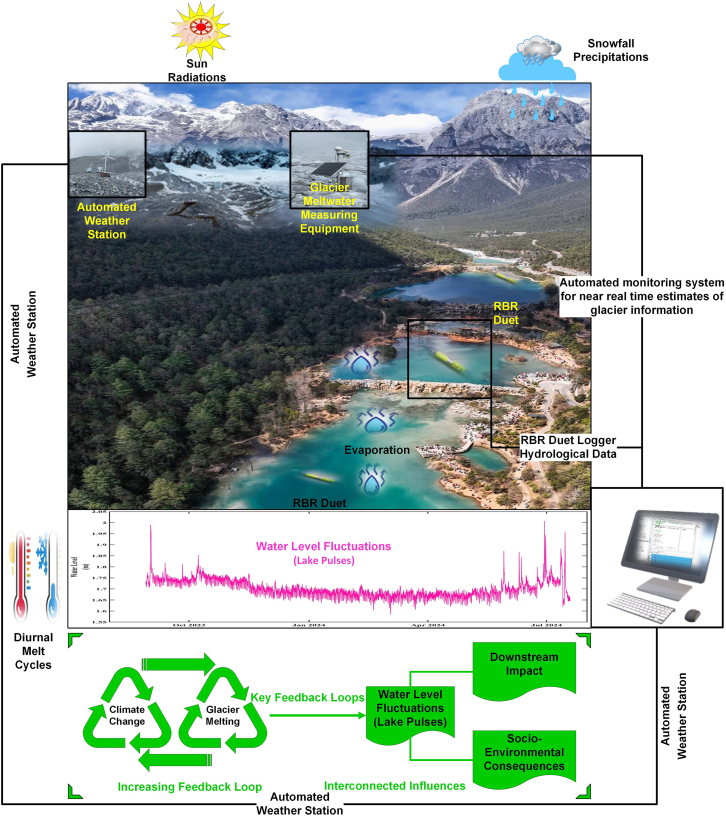
Conceptual scalable framework illustrating climate-cryosphere-hydrology interactions and their impacts on glacial lakes dynamics

To extend the applicability of this approach across glacierized basins in the TP and other high-altitude regions, we propose this scalable and transferable framework for analyzing glacier-lake interactions under contemporary climate forcing (Figure 7). This framework integrates GBM-based glacier melt modeling, high-resolution hydrological monitoring, and systematic climatic attribution. It is adaptable to varying data infrastructures: in well-instrumented basins, it can leverage automatic weather stations and satellite reanalysis products (e.g., ERA5, CHIRPS, MODIS); in data-sparse regions, it supports regional transferability through parameter scaling based on glacier hypsometry, elevation bands, and climatic zonation.

The framework presented in (Figure 7) also incorporates continuous lake-level monitoring using *in-situ* sensors, with observations decomposed into trend, seasonal, and event-scale components. This enables the attribution of lake-level changes to specific drivers—such as heatwaves, monsoon bursts, or anomalous snowmelt—through correlation and lag analysis. Evaporation losses, often under-constrained in lake water balance studies, are estimated using physically based methods (e.g., Penman or Priestley-Taylor), depending on data availability.^25^ These estimates improve the partitioning of net lake-level changes into glacier-derived inputs, rainfall recharge, and atmospheric losses. The comparative scaling framework (Table 2) highlights how variations in glacier contribution, climate regime, and elevation influence lake sensitivity.^26^^,^^27^^,^^28^ Although the framework was not directly applied to Qinghai Lake, Nam Co, or Ranwu Lake, these systems were included as contextual benchmarks to illustrate the diversity of glacial lake climate interactions across the TP. Their inclusion supports the conceptual scalability of the framework, even though empirical validation was not conducted for these specific sites. For example, Blue Moon Lake Valley shares similarities with proglacial lakes such as Ranwu Lake, where high glacier melt contributions (>50%) and monsoon influence drive pronounced seasonal hydrological variability. In contrast, terminal lakes like Qinghai Lake, with low glacier inputs (∼5–10%) and cold-arid climates, exhibit reduced sensitivity to glacial processes and greater dependence on precipitation-evaporation balances.^29^^,^^30^ Nam Co, situated in a transitional climatic zone, further exemplifies how lake response can vary with both elevation and monsoonal intensity. These comparisons reinforce that monsoon-dominated glacial lakes at lower elevations are especially vulnerable to rapid hydrological changes, while larger continental lakes may exhibit more inertia but are not immune to long-term drying trends.

**Table 2:** Comparative scaling table for QTP glacial lakes

Parameter	Blue Moon Lake Valley	Qinghai Lake	Nam Co Lake	Ranwu Lake
Elevation (m)	∼3200 m	∼3194 m	∼4718 m	∼3850 m
Lake type	Proglacial	Terminal	Semi-closed	Proglacial
Glacier contribution (%)	High (60–87%)	Low (∼5–10%)	Medium (∼30%)	High (>50%)
Evaporation rate (mm/year)	Moderate (∼700–1000)	High (∼930)	Moderate (∼700)	Low (∼500)
Dominant climate	Monsoonal subtropical	Cold-arid continental	Monsoonal alpine	Monsoonal alpine
Melt-driven sensitivity	Very high	Low	Moderate	High

The increasing dominance of evaporation relative to glacier melt marks a critical inflection point for lake stability across the region. Progressive glacier retreat, combined with rising temperatures, could lead to declining lake levels, reduced water availability, and heightened risks of glacial lake outburst floods.^20^^,^^31^^,^^32^^,^^33^ This draws attention to the need for predictive tools that integrate cryosphere-hydrosphere coupling, as demonstrated by,^13^^,^^34^ to support basin-specific forecasting and risk mitigation strategies.^35^ Finally, the modular design of the proposed framework allows for adaptation to diverse glacier geometries, climate regimes, and data conditions. As^36^ emphasized, modeling glacier-lake systems requires accounting for spatial heterogeneity, data scarcity, and dynamic feedback. Our framework responds to this challenge by combining physically interpretable machine learning with high-resolution monitoring and lag analysis, offering a robust, transferable tool for advancing cryosphere–hydrology research across High Mountain Asia.

### Limitations of the study and future directions

While this study presents a high-resolution analysis of melt-lake dynamics, certain limitations must be acknowledged. The influence of groundwater fluxes was not explicitly quantified, potentially contributing to some of the observed lag effects between melt inputs and lake water level responses. Future work should prioritize expanding the temporal scope of observations across multiple seasons and years to capture broader climatic variability. Moreover, coupling *in situ* measurements with remote sensing estimates of glacier mass balance and lake surface area changes (using MODIS, Landsat, and Sentinel imagery) would enhance the robustness of water source attributions. Application of this framework across additional glacial lakes on the TP, with varying climatic settings (monsoon-dominated vs. westerly-dominated), would further clarify the degree to which the processes identified here are basin-specific or broadly generalizable across High Mountain Asia. Ultimately, integrating hydrological modeling, remote sensing, and field measurements offers the most promising pathway to improve predictive understanding of glacial lake dynamics under ongoing climate change.

## Resource availability

### Lead contact

Further information and requests for resources should be directed to and will be fulfilled by the lead contact, Songtao Ai (ast@whu.edu.cn).

### Materials availability

All the materials of this study are available from the lead contact without restriction and can be provided upon reasonable request.

### Data and code availability

• Glacier melt rates, AWS data, and lake-level measurements used in this study are available from the lead contact upon reasonable request.
• Custom code used for glacier melt modeling, cross-correlation analysis, and feature importance evaluation is available from the lead contact upon request.
• Additional information regarding the data and analyses presented in this study is available from the lead contact upon request.

## Acknowledgments

This study was supported by the 10.13039/501100012166National Key R&D Program of China (2024YFC2816301) and the 10.13039/501100012226Fundamental Research Funds for the Central Universities of China (2042025kf0078).

## Author contributions

Conceptualization, S.A.S. and S.A.; methodology, S.A.S., investigation, S.A.S. and S.A.; writing—original draft, S.A.S.; writing—review & editing, S.A.S. and S.A.; funding acquisition, S.A.; resources, S.A., and supervision, S.A.

## Declaration of interests

The authors declare that they have no known competing financial interests or personal relationships that could have appeared to influence the work reported in this paper.

## STAR★Methods

### Key resources table

**Table:** 

REAGENT or RESOURCE	SOURCE	IDENTIFIER
**Deposited data**		

Lijiang Meteorological Station	China Meteorological Data Service Center	http://data.cma.cn

**Software and algorithms**		

MATLAB R2025a	MathWorks	https://www.mathworks.com
Ruskin 2.23.1	RBR	https://rbr-global.com
ArcGIS 10.8.3	Esri	https://www.esri.com

### Method details

#### Yulong Snow Mountain glacier system and Blue Moon Lake Valley

The study was conducted in the southeastern sector of the QTP, focusing specifically on the YSM glacial system and its associated proglacial Blue Moon Lake Valley. This region presents a unique setting to investigate high-frequency glacier-lake-climate interactions due to its steep topographic gradients, maritime climatic influences, and rapid Cryospheric changes. YSM is situated between 27.16°–27.66°N latitude and 100.15°–100.33°E longitude, within Yunnan Province, China (Figure S1). It represents the southernmost extent of temperate glaciers on the Eurasian continent. The mountain stretches approximately 35 km from north to south and 13 km from east to west, with its highest peak, Shanzidou, reaching 5,596 m above sea level. As of the most recent comprehensive survey in 2017, YSM hosts 13 glaciers covering a total area of approximately 4.48 km^2^. These glaciers are characterized by low accumulation zones, ranging from 5,361 m down to termini at about 4,395 m.^37^ The largest glacier, Baishui River Glacier No. 1, spans approximately 1.32 km^2^ and has shown pronounced retreat and thinning over the past few decades. Observations from meteorological stations at the mountain’s base indicate a significant warming trend of 0.3°C per decade between 1980 and 2013, contributing to enhanced glacial ablation and mass loss. The glacier system lies within a critical climatic transition zone influenced by the interplay of the Indian summer monsoon, the Southeast Asian monsoon, and the southern branch of the westerlies. These climatic dynamics drive substantial interannual variability in precipitation and energy fluxes, which in turn regulate the glacier’s meltwater production.

The climate of the YSM region is strongly influenced by the Southeast Asian monsoon, which delivers most of the annual precipitation between June and September. Annual precipitation at lower elevations around Lijiang averages 1,000–1,200 mm, decreasing with elevation. The summer monsoon brings both intense rainfall events and elevated air temperatures, amplifying glacier surface melt through enhanced sensible heat fluxes and incoming shortwave radiation. Outside the monsoon season, the region experiences drier conditions dominated by the southern branch of the mid-latitude westerly, particularly during winter and early spring. These seasonal atmospheric patterns create strong energy and mass balance gradients, leading to highly variable glaciers and lake dynamics over sub-seasonal to diurnal timescales. The combination of rapid glacier retreat, strong monsoonal modulation, and dynamically evolving lake systems underscores the importance of this region as a sentinel of broader cryospheric and hydrological transformations occurring across high-mountain Asia.

Approximately 10 km downstream from Baishui River Glacier No. 1 lies Blue Moon Lake Valley, positioned at an elevation of approximately 3,200 m above sea level (27° 07'44"N, 100° 14'37" E). The lake is a proglacial body formed by meltwater inflow, accounting for an estimated 60–75% of its hydrological input during the ablation season. Blue Moon Lake Valley exhibits typical characteristics of glacial alpine lakes, including pronounced thermal stratification during summer months, with epilimnion temperatures reaching between 12–18°C and hypolimnetic layers maintaining colder temperatures around 4–6°C. Water level fluctuations within the lake show distinct diurnal and seasonal patterns, closely linked to variations in glacial melt rates, precipitation events, and evaporative losses. The lake is not only significant for hydrological and Cryospheric studies but also holds ecological and socio-economic importance, serving as a tourist destination and a freshwater resource for surrounding communities. Its dynamic response to glacial and climatic forcings makes it an ideal site for analyzing fine-scale hydroclimatic variability in a rapidly warming environment.

#### Data sources

A multi-source observational dataset was employed to comprehensively investigate the hydroclimatic dynamics of the Baishui River Glacier No. 1 and its associated Blue Moon Lake Valley. These datasets cover glaciological, hydrological, and meteorological variables at fine temporal resolutions, enabling high-fidelity analysis of glacier–lake–climate interactions.

##### Hydrological data

Continuous high-frequency measurements of water level, pressure, and temperature were obtained using RBR Duet pressure–temperature loggers. Data extraction and preprocessing were performed using Ruskin 2.23.1 software. The sensors, deployed within the lake, recorded measurements at 5-minute intervals. Before deployment, all sensors were calibrated. Subsequent quality control procedures included the removal of spurious spikes and corrections for atmospheric pressure fluctuations using local barometric data. For this study, the raw measurements were aggregated to an hourly resolution to balance data fidelity and noise reduction.^5^ The monitoring period spanned from August 2023 to July 2024, covering the late ablation season when glacier meltwater contributions were most pronounced (Figure S3A).

##### Glacier and meteorological data

Glaciological parameters were sourced from a combination of *in situ* observations. Direct observations of glacier surface depth and ice thickness at Baishui River Glacier No. 1 were collected during the summer field campaigns of 2023 (Figure S2). The meteorological variables were sourced from two complementary datasets to accurately capture atmospheric forcing conditions over the study area. Lijiang Meteorological Station Data, located approximately 3 km away from Blue Moon Lake Valley at Lijiang City, provided continuous records of air temperature, solar radiation, relative humidity, atmospheric pressure, precipitation, and wind speed.^13^^,^^34^ This national-level station recorded data at hourly intervals, with a high degree of reliability and long-term calibration histories. Supplementary meteorological observations were collected from the YSM on-site station (Figure S2), a dedicated automatic weather station installed near Baishui River Glacier No. 1 at an elevation exceeding 4,000 m.^5^^,^^38^ This station provided localized data on glacier surface conditions, including near-surface air temperature, incoming shortwave radiation, and precipitation during the ablation period (Figures S3B–S3H). The combination of these two data sources enabled the interpolation of climatic conditions across the elevational gradient from glacier accumulation zones to the lake basin, ensuring realistic input for glacier melt modeling and hydroclimatic correlation analyses.

#### Methodological overview

This study employed high-frequency (hourly) hydro-meteorological observations from August 2023–July 2024 to investigate glacier-lake dynamics in the Baishui River Glacier No.1 and Blue Moon Lake Valley system. Key variables monitored included lake water levels, air temperature, solar radiation, rainfall, evaporation rates, pressure, humidity, and wind speed. A GBM model was applied to simulate glacier meltwater production, in addition to past and future forecasts, validated through correlation with observed and modelled melt rate. Time-series decomposition and cross-correlation analyses were used to attribute water level fluctuations to glacier melt, precipitation, and evaporation inputs. The integrated approach enabled the quantification of meltwater contributions and hydrological response delays, providing a high-resolution framework for understanding cryosphere–hydrosphere interactions in monsoon-affected, high-altitude basins. Furthermore, (Figure S4) a flow chart diagram illustrates the overall methodology.

#### Glacier melt modeling approach using GBM

In this study, glacier melt dynamics in the Baishui River Glacier No. 1 and Blue Moon Lake Valley were simulated using a data-driven approach based on the GBM algorithm. Unlike traditional empirical temperature-index models such as the degree-day method, which assume a linear relationship between air temperature and melt rates,^19^^,^^39^ GBM gives a robust framework for capturing complex, nonlinear interactions among multiple meteorological drivers.^9^^,^^13^ The GBM model was trained on an hourly dataset consisting of observed glacier melt values and corresponding meteorological predictors. Key input variables included air temperature, solar radiation, and rainfall, along with derived interaction terms such as temperature × solar radiation and temperature × rainfall. To incorporate temporal variability, features such as hour of day, day of month, month, year, weekday, and seasonal indicators (spring, summer, autumn, winter) were also included.

Before model development, feature engineering was performed to ensure that the dataset captured relevant physical interactions and temporal structures. Outliers in meteorological variables were treated using the interquartile range (IQR) method to enhance model robustness, following the data preprocessing procedures recommended by.^13^ The model was implemented in MATLAB R2025a using the fitrensemble function with the LSBoost method, comprising 200 learning cycles and regression trees with a maximum of 20 splits as base learners. The dataset was split into 80% for training and 20% for validation and testing.^34^ Model performance was evaluated using standard statistical metrics, including root mean square error (RMSE) and mean absolute error (MAE). The GBM model exhibited high predictive accuracy, successfully capturing both the magnitude and temporal patterns of observed glacier melt. In addition to predictive modeling, the trained GBM was used to generate synthetic past and future melt scenarios by simulating meteorological inputs based on historical distributions. Notably, predictions for both past and future conditions were generated for the same temporal window as the observed dataset to ensure consistency in comparative assessments. By utilizing the ability of GBM to model nonlinear relationships and interactions, this approach provides a more adaptive and physically meaningful alternative to traditional glacier melt models.^5^^,^^40^ Recent research has also supported the use of GBM and other machine-learning algorithms for hydrological and Cryospheric modeling , particularly in glacierized catchments.^41^^,^^42^

#### Water balance components and data processing

To identify the primary drivers of lake water level fluctuations, we analyzed three key hydrological fluxes: glacier meltwater input, direct precipitation, and evaporation losses. Hourly time series data for each component were used to capture high-resolution temporal variability. Water level changes were calculated as the difference between consecutive hourly measurements and converted from meters to millimeters for consistency and precision (Equation 1). This metric served as the response variable in subsequent attribution and statistical analyses.

(Equation 1) $$\Delta \text{WL}\;\left(\text{mm}\right)=1000\times \left({\text{WL}}_{t}-{\text{WL}}_{t-1}\right)$$

Where: ΔWL(mm) = hourly change in lake water level in millimeters, ${\text{WL}}_{t}$ = lake water level at the current time step t (meters) and ${\text{WL}}_{t-1}$= lake water level at the previous time step t−1 (meters). This hourly water level change metric served as the response variable in subsequent attribution and statistical analyses.

##### Attribution of hydrological inputs

The net hydrological input influencing lake level changes was defined in Equation 2. Where glacier melt and rainfall represent positive inputs, and evaporation denotes water loss.

(Equation 2) ExplainedChange=GlacierMelt+Rainfall−Evaporation

To quantify the relative contributions of glacier melt and rainfall to positive inflows, their proportions were calculated by (Equations 3 and 4).

(Equation 3) $${\text{Glacier}\;\text{Melt}}_{\text{contribution}}=\frac{\text{Glacier}\;\text{Melt}}{\text{Glacier}\;\text{Melt}+\text{Rainfall}}$$

(Equation 4) $${\text{Rainfall}}_{\text{contribution}}=\frac{\text{Rainfall}}{\text{Glacier}\;\text{Melt}+\text{Rainfall}}$$

Furthermore, cumulative volumes for each flux were obtained by summing hourly rates (Equations 5, 6, and 7) across the observation window:

(Equation 5) $$\text{Glacier}\;\text{Melt}\;\text{Volume}=\sum_{t}{\text{Melt}\;\text{Rate}}_{t}$$

(Equation 6) $$\text{Precipitation}\;\text{Volume}=\sum_{t}{\text{Rainfall}\;\text{Rate}}_{t}$$

(Equation 7) $$\text{Evaporation}\;\text{Volume}=\sum_{t}{\text{Evaporation}\;\text{Rate}}_{t}$$

Total flux was computed as the sum of absolute contributions from all processes, with relative percentages expressed in Equation 8.

(Equation 8) $$\text{Contribution}\;\left(\%\right)=\frac{\text{Cumulative}\;\text{Volume}\;\text{of}\;\text{Component}\;\text{Sum}\;\text{of}\;\text{Absolute}\;\text{Volumes}}{\text{Sum}\;\text{of}\;\text{Absolute}\;\text{Volumes}}\times 100$$

#### Statistical modeling and variable importance

A random forest regression model was applied to assess the predictive importance of glacier melt, rainfall, solar radiation, humidity, wind speed, and evaporation on lake-level changes. Hydrological variables were normalized via min-max scaling before training. Feature importance was derived using out-of-bag error permutation, providing a robust measure of each variable’s contribution to model performance^5^^,^^13^ for detailed methodology.

#### Cross-correlation and lag analysis

To evaluate the temporal relationships between hydrological inputs and lake-level fluctuations, the Pearson correlation coefficient (Equation 9) was computed between the explained water level change-defined as the net sum of glacier melt, rainfall, and evaporation and observed hourly water level differences.^43^^,^^44^ This approach quantifies the linear association between modeled and observed dynamics.

(Equation 9) $$r=\frac{\sum_{i=1}^{n}\left(X_{i}-\bar{X}\right)\left(Y_{i}-\bar{Y}\right)}{\sqrt{\sum_{i=1}^{n}{\left(X_{i}-\bar{X}\right)}^{2}}\sqrt{\sum_{i=1}^{n}{\left(Y_{i}-\bar{Y}\right)}^{2}}}$$

Where n is the number of valid hourly observations, $X_{i}$ and $Y_{i}$ are the paired observations of explained change and observed water level differences, and $\bar{X}$ and $\bar{Y}$ are their respective means. To account for potential lagged hydrological responses due to hydrodynamic buffering, cross-correlation analyses was conducted with temporal lags up to 24 hours. This lag structure allowed the detection of delayed lake responses to glacier melt and precipitation inputs, thereby improving the temporal attribution accuracy.

#### Hourly peak analysis and glacier melt–lake response timing

To investigate the temporal relationship between glacier meltwater production and lake water level fluctuations, a systematic peak detection and time lag analysis was performed based on high-frequency (hourly) datasets. First, peaks were identified separately in the time series of modelled glacier melt rates and observed lake water levels. Local maxima were detected using a prominence-based method, ensuring that each extracted peak represented a significant hydrological event rather than noise. For both variables, peaks were further classified into first, second, and third occurrences within each day, enabling daily characterization of hydrological pulse intensities. Subsequently, the timing of successive peaks was analyzed. The temporal differences between consecutive peaks were calculated to determine the frequency and regularity of meltwater and water level pulses.^5^ Average time intervals were computed for the first, second, and third peaks individually to reveal any diurnal or sub-daily patterns in hydrological dynamics.

To quantify the direct response time of the lake system to upstream meltwater inputs, a cross-peak timing analysis was conducted. Each identified glacier melt peak was matched to the nearest subsequent water level peak. Given the known spatial separation between the glacier and lake (∼10 km), an assumed mean meltwater flow velocity of 5 km/hour was applied to correct for physical transit delay, yielding an expected lag time of approximately 2 hours. The residual time lag between corrected glacier melt peaks and lake level peaks was then calculated and averaged across all matched events. This approach provided a first-order estimation of the meltwater routing time and system response delay under current glaciological and hydrological conditions. This peak-based approach allowed a detailed examination of how short-term glacier melt events influence lake-level changes, helping to understand the timing and connection between glacial runoff and lake response under changing weather conditions.

### Quantification and statistical analysis

All data preprocessing, modeling, and statistical analyses were performed in MATLAB R2025a (https://www.mathworks.com) following the extraction of raw hydrological measurements from RBR loggers using Ruskin 2.23.1 (https://rbr-global.com). The dataset, comprising approximately 7,806 hourly observations per variable, provided high temporal resolution. Descriptive statistics—including mean, median, standard deviation, minimum, maximum, and interquartile range—were computed for each parameter and are summarized in Table 1. Key meteorological variables and model outputs are visualized in Figures 1 and S3. Correlation patterns and model performance metrics are detailed in the results section and illustrated in Figures 1, 2, 3, 4, 5, and 6. Feature importance scores from a GBM (LSBoost) were used to evaluate the predictive influence of individual climatic variables and their interactions (temperature × solar radiation, temperature × rainfall) on glacier melt (Figures 1 and 3). Model performance was evaluated using RMSE and MAE to assess the accuracy of glacier melt simulations (Figure 2A). To examine hydrological connectivity, peak-based analysis (findpeaks) was applied to glacier melt and lake depth time series to identify significant events. Time-lag correlations between glacier melt peaks and downstream lake-level responses were quantified, yielding an average lag-adjusted response time (Figure 4). To quantify the relative contribution of glacier melt and climatic factors to lake-level variability, a Random Forest regression model was applied to min-max scaled data. Out-of-bag predictor importance scores revealed the relative influence of each variable on observed lake depth (Figure 5). Pearson correlation coefficients (r) were used to quantify relationships among glacier melt, net hydrological inputs (glacier melt + rainfall − evaporation), and observed lake depth fluctuations (Figure 6). In this context, n denotes the number of valid paired hourly observations used in the calculation, where each pair consists of simultaneous measurements of the two variables.
