# Supplementary material for: High-resolution modeling of glacier meltwater contributions to lake water level fluctuations in the Baishui River Glacier No.1 basin
Source: iScience. 2025 Aug 7;28(9):113321. doi: 10.1016/j.isci.2025.113321 (PMC12496220; doi:10.1016/j.isci.2025.113321)
Supplement: Document S1. Figures S1–S4 [file mmc1.pdf]

iScience, Volume *28*

Supplemental information

High-resolution modeling of glacier meltwater contributions to lake water level fluctuations in the Baishui River Glacier No.1 basin

Shoukat Ali Shah and Songtao Ai

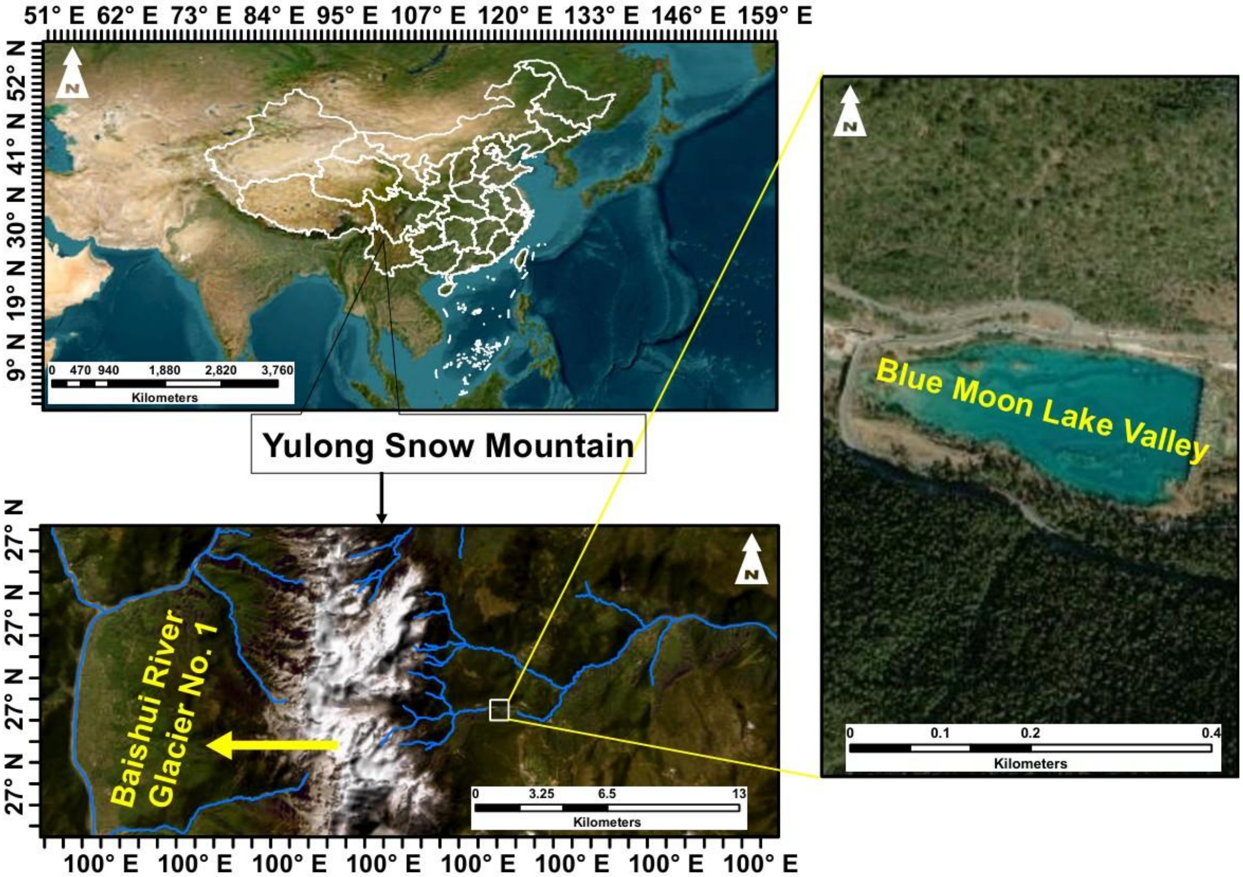

Figure S1. Layout map of Blue Moon Lake Valley and Baishui River Glacier No.1 at Yulong Snow Mountain, Yunnan Province, China, (Map prepared using ArcGIS 10.8.3)

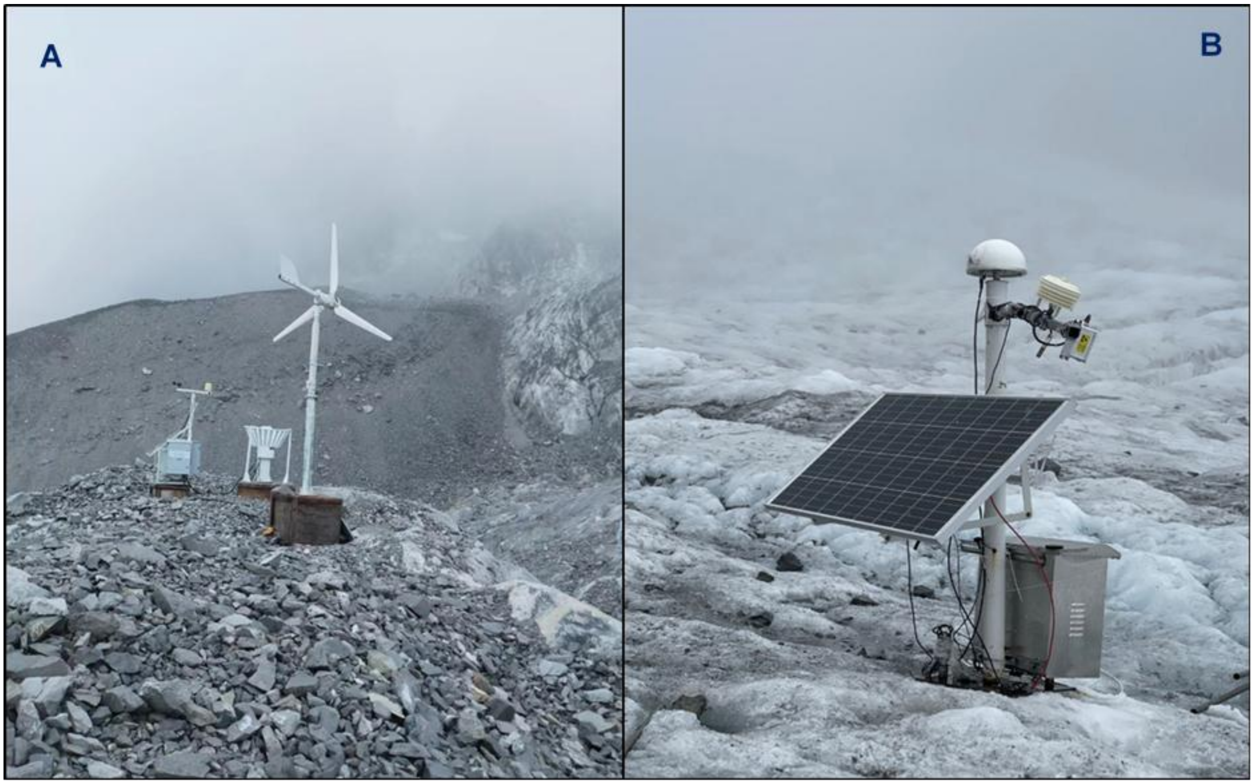

Figure S2. Automatic weather station setup at Baishui River Glacier No. 1, Yulong Snow Mountain.
(A) Instruments on rocky terrain for atmospheric monitoring
(B) Solar panel and equipment on glacier ice for glacier observations.
(Source: Photograph by the first author)

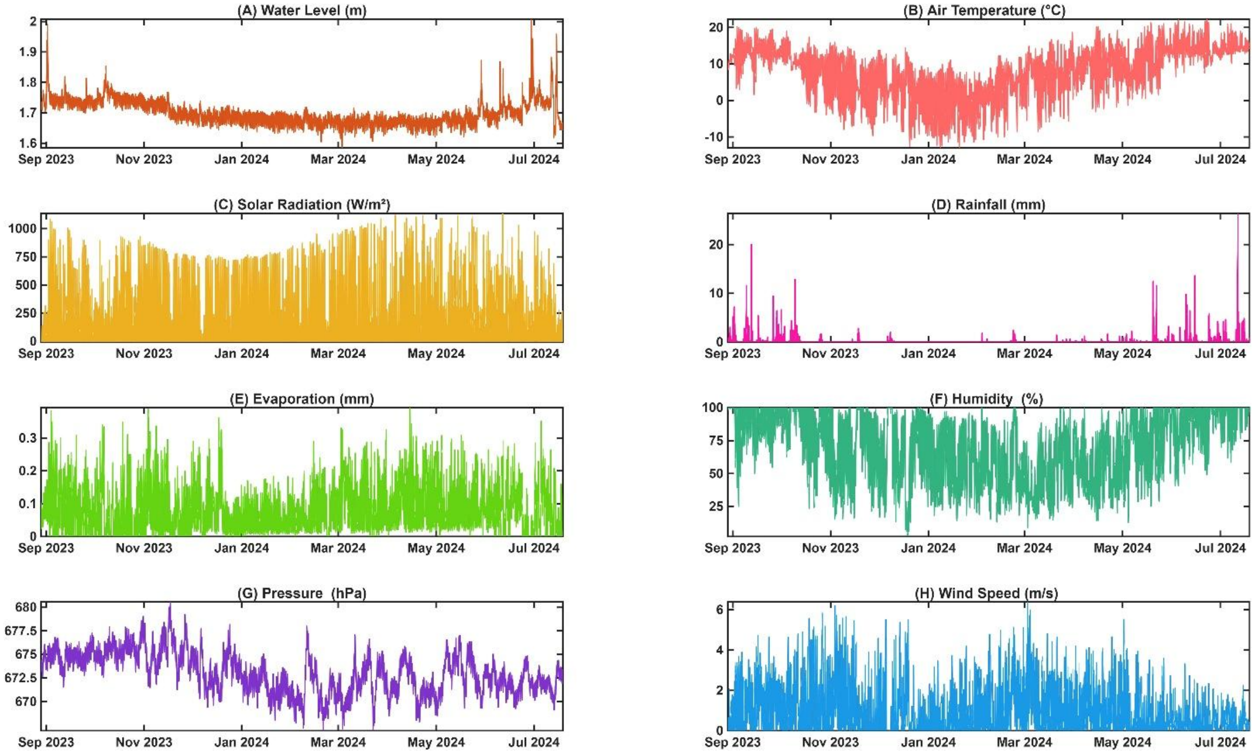

Figure S3. High-resolution hydro-meteorological observations records collected at study site.
(A) Water level
(B) Air temperature
(C) Solar radiation
(D) Rainfall
(E) Evaporation
(F) Humidity
(G) Pressure
(H) Wind Speed

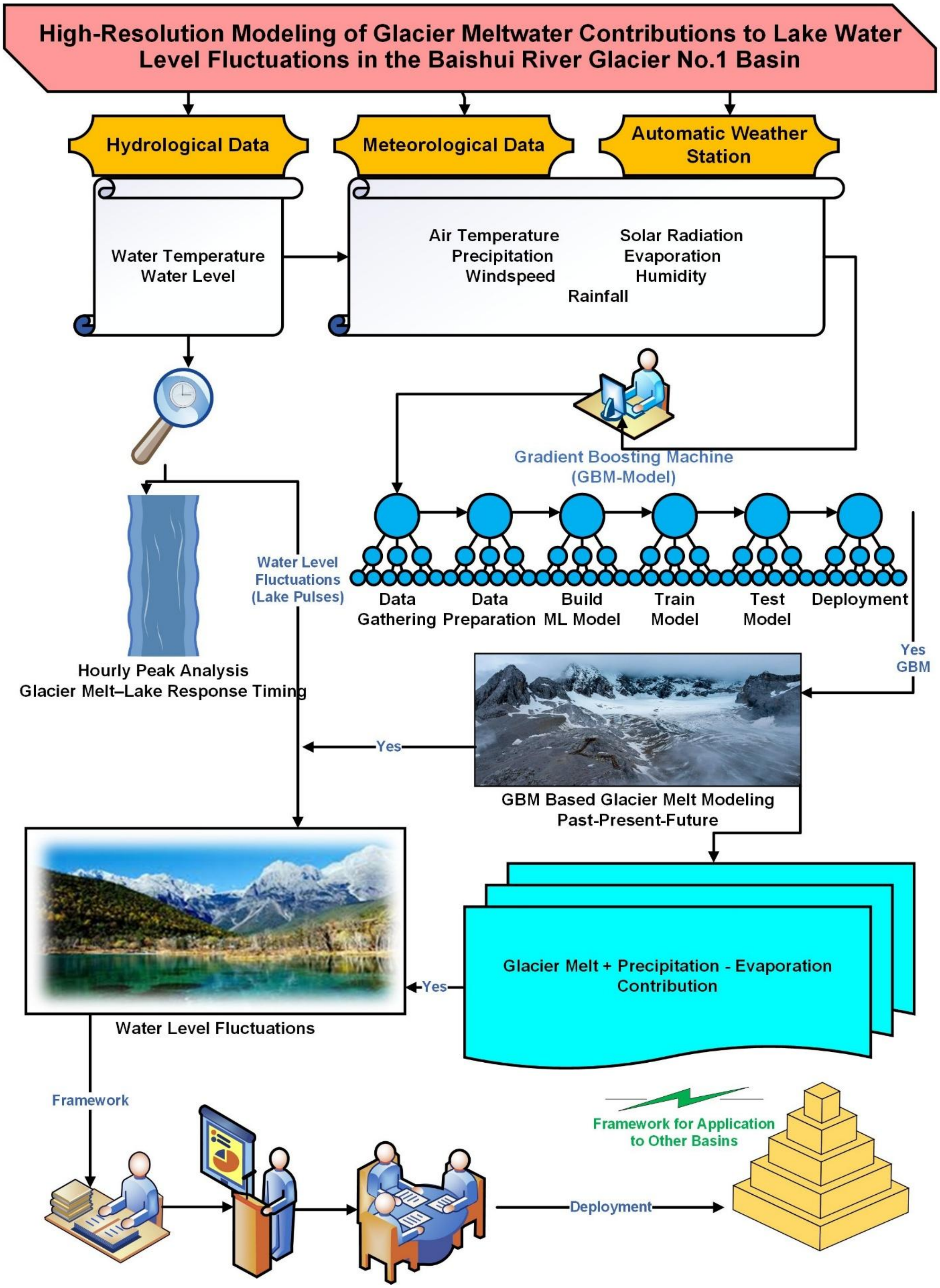

Figure S4. Flowchart diagram for this study
